# Metabolism and gene sequence variation in *Turicella otitidis* implies its adaptability and pathogenicity in extra-otic infection: a systematic review

**DOI:** 10.1186/s12879-023-08721-y

**Published:** 2023-10-27

**Authors:** Afrinash Ahamad, Cuishan Yuan, Casey Chung, Briana Blair, Amy Tran, Bushra Tehreem

**Affiliations:** 1 Clinical Laboratory Sciences Program, School of Health Profession, Stony Brook University, Stony Brook, NY USA; 2 Department of Neuroscience and Behavior, Stony Brook University, Stony Brook, NY USA; 3 Department of Pathology, Clinical Microbiology, NYU Langone Health, New York, NY USA; 4 Department of Pediatrics- SUNY Down State, Brooklyn, NY USA

**Keywords:** *Turicella otitidis*, Otitis media, Extra-otic, *Corynebacterium*

## Abstract

*Turicella otitidis* belongs to the Corynebacteriaceae family and is a normal inhabitant of the ear and exists in a commensal relationship with its host. In children, *T. otitidis* is frequently associated with otitis media. The emergence of *Turicella otitidis* as a pathogen is concerning, particularly due to the limited availability of data on its pathogenic properties. The objective of this study is to conduct a systematic review of *T. otitidis* infections occurring in both the ear and other anatomical sites, and to summarize the differences in metabolism and genome sequences between isolates obtained from the ear and blood.

**Supplementary Information:**

The online version contains supplementary material available at 10.1186/s12879-023-08721-y.

## Introduction

*Turicella otitidis* is a Gram-positive *Corynebacterium* that grows aerobically [1]. It is commonly part of normal ear resident flora, although it is frequently linked to external ear canal infection and acute and chronic otitis media in children [2]. Recent reports showed that the organism can cause extra-otic infections such as microbial keratitis, bacteremia, and posterior auricular abscess [3] in patients with or without underlying conditions, indicating its pathogenicity. In a diagnostic microbiology laboratory, *T. otitidis *is often not distinguished from commensal skin coryneform isolates. Depending on the source of the specimen and the number of specimens sent for culture, such as blood culture, the organism is reported as “*Corynebacterium* species, not *C. jeikeium* or *C. striatum*”. Nevertheless, advanced methodologies such as Matrix-Assisted Laser Desorption/Ionization Mass Spectroscopy (MALDI-TOF MS) have facilitated the accurate and rapid speciation of *Corynebacterium* [4]. Despite this, the traditional API Coryne method remains fundamental to the identification process, and further biochemical reactions, such as catalase, CAMP, DNase positive reaction, and oxidase negative reaction, aid in the identification of *Turicella otitidis*. *Corynebacterium* species are commonly treated with penicillin, macrolides, rifampin, vancomycin, and fluoroquinolones. Notably, *T. otitidis* exhibits resistance towards sulfamethoxazole, cotrimoxazole, and macrolide [5]. Thus, accurate identification and speciation are imperative for optimal treatment.

Since *T. otitidis* has traditionally been deemed non-pathogenic [6], little attention has been paid to its clinical relevance. Recent reports of extra-otic infections warrant the need to study the factors associated with the organism’s pathogenicity and adaptability in various niches. As the genome code and metabolism are crucial for all biological processes, including virulence, it is imperative to examine the factors and mechanisms underlying the virulence of *T. otitidis*. We herein summarized the information on the metabolism and genome sequence variation in *Turicella otitidis* isolated from two distinct sources, namely the ear and blood, as well as cases of otic- and extra-otic infection resulted from *T. otitidis*.

### Objective

*Turicella otitidis* is frequently involved in both acute and chronic otitis media. Its adaptability and isolation from extra-otic infections make it an emerging pathogen. This study aims to understand the niche-specific modifications in *T. otitidis* metabolism and genome sequences and its role in pathogenesis in extra-otic infections compared to ear infections.

## Methods

We conducted a comprehensive search of the literature by utilizing multiple databases, including PubMed, Science Direct, Cochrane, EMBASE, CINAHL, and Google Scholar. Our search was focused on identifying pertinent cases of *T. otitidis* infection, both within the otic and extra-otic domains, spanning from 1994 to 2023. Two authors independently screened all abstracts resulting from the initial literature search, with duplicate and non-pertinent articles subsequently removed. Quality assessment was conducted to ensure that the included articles solely focused on the association or discussion of *Turicella otitidis* concerning infection in either the ear or other sources. To filter the articles, keywords such as “*Turicella otitidis*”, “*T. otitidis*”, “*Turicella otitidis* metabolism”, “*Turicella otitidis* genome sequences”, and “*T. otitidis* genome sequence and metabolism” were used (Electronic Supplementary file, S1). Articles that mentioned *T. otitidis* in the abstract or studies in which only ear specimens were analyzed for their microbiota and their association with ear infections were included. Exclusion criteria included duplicate and extraneous articles and papers other than case reports and retrospective studies. The search was not restricted to regions and languages, but solely to publication types. The relevance and accuracy of the articles were carefully assessed after inclusion and exclusion parameters were established. A total of seven articles were in non-English languages (NEL), specifically Spanish (5), Czech (1), and French (1). To determine the eligibility of the articles, Google Translate was initially utilized to analyze their abstracts. Eligible articles were subsequently translated by volunteers, including students, researchers, and healthcare professionals. The Stony Brook University Language Department and Clinical Pathology specifically Clinical Microbiology and Hematology departments were solicited for volunteers. The translators proofread and edited the text.

## Results

**Fig. 1:**
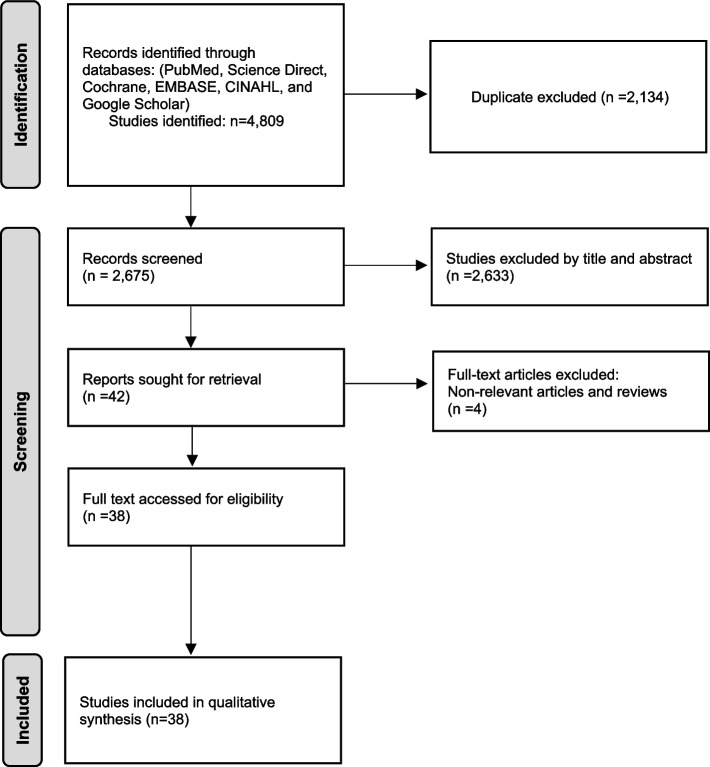
PRISMA flowchart of literature search and inclusion process of studies. Adapted from: Page MJ, McKenzie JE, Bossuyt PM, Boutron I, Hoffmann TC, Mulrow CD, et al. The PRISMA 2020 statement: an updated guideline for reporting systematic reviews. BMJ 2021

A total of 4809 articles were identified from PubMed, Science Direct, Cochrane, EMBASE, CINAHL, and Google Scholar databases. After excluding duplicates (*n* = 2134) and non-pertinent studies (*n* = 2633) from the thorough article evaluation, a full-length review was performed on the eligible articles (*n* = 42) and additional articles (*n* = 4) that did not meet the inclusion criteria were excluded. A comprehensive full-text assessment of the articles was thoroughly conducted according to the PRISMA flow chart [7] (Fig. 1). In total, thirty eight articles were included in the study and two of these studies compared the metabolism changes and genome variation in *T. otitidis* isolated from two sources (ear and blood). Out of 36 articles, 52.7% (19 articles) were case studies (Table 1) and 47.2% (17 articles) were retrospective studies (Table 2) [1–4, 8–39]. Of the total studies, 81.5% of the articles were in English, while 18.4% were in NEL. Based on the case description, thirteen cases (54.2%) of *T. otitidis* infection were in males while eleven cases (45.8%) were in females. Of the total 24 cases, ages ranged from 6 months to 75 years, 37.5% of the cases were reported in age < 5 years (male = 16.7%, female = 20.8%), while 29.2% were among individuals aged 5–15 years (male = 16.7%, female = 12.5%). Furthermore, 8.3% of cases were reported in the age group of 20–35 years (male = 4.17%, female = 4.17), and an equal percentage of 8.3% was seen in the age group 40–55 years of age (male = 8.33%, female = 0). Lastly, 16.7% of the cases were reported in individuals > 55 years of age (male = 8.33%, female = 8.33%). Of the total case reports, 16 cases (66.7%) were associated with ear infections, 4 cases (16.7%) were attributed to a bloodstream infection, and 2 cases (8.3%) were associated with ocular infection. Moreover, 4.2% of the cases were linked to skin and 4.2% were associated with abscesses in the cervical region. In light of the outcome of these cases, all (100%) of the patients survived with improvement in symptoms post-treatment. Of the total twenty four cases, 50% used a combination of various techniques including API Coryne, Vitek MS, biochemical analysis, microcopy, culture, Rapid CB Plus kit, High-performance liquid chromatography (HPLC), and MALDI MS for identification of *T. otitidis*. Meanwhile, the remaining 50% used a single method of identification such as Culture or MALDI-TOF MS or PCR. Of the total retrospective studies, 52.9% used multiple modalities, including VITEK MS, Microscan Panels (PC42 HIND), API Coryne, API Zym, API 50CH, as well as morphological and phenotypic tests such as CAMP test, DNASE test in the identification process. On the other hand, 41.2% relied solely on a single method such as 16S rRNA sequencing, DNA sequencing, Immunoblot DNAB II proteins, Western blot and 16S metagenomics, for the identification of *T. otitidis*. Additionally, in one study (5.9%), method of identification was not specified. The common treatment regime was amoxicillin-clavulanic acid (25%), and vancomycin (12.5%). In 20.8% of the cases, topical treatment was used. Additionally, the utilization of amoxicillin, fosfomycin, penicillin, rifampin, gentamycin, cefotaxime, and ciprofloxacin was reported in 41.7% of the cases. These antibiotics were administered either as monotherapy or given in combination [1–4, 8–39].

**Table 1:** Shows the summary of *Turicella otitidis* case reports. NS = not specified, Polymerase Chain Reaction (PCR)

No.	Author & year	Age & sex	Condition	Source of isolation	Method of identification	Treatment
1	Mastroianni, A., et al., 2023 [40]	11-year old, male	Acute lymphoblastic leukemia with acute or chronic otitis, mastoiditis, sinusitis	Central venous catheter (CVC) blood	Direct microscopy, Vitek2-Vitek MS	Intravenous rifampicin, vancomycin CVC lock therapy, and CVC removal
2	Priyadarshini, S.R., et al.,2021 [36]	10-year-old, male	Microbial keratitis	Corneal graft	VITEK® 2 automated system	Topical gatifloxacin 0.5% eye-drops
3	Koumaki, D., et al., 2020 [33]	74-year-old, female	Palmoplantar eczema	Skin	VITEK® 2 automated system and biochemicals	Cefuroxime
4	Mammo, D.A., D. Watson, and K.R. Armbrust,2020 [26]	71-year-old, male	Neovascular age-related macular degeneration	Endophthalmitis	PCR	NS
5	Li, D., et al., 2019 [11]	69-year-old, female	Diffuse large B cell lymphoma, fever	Blood	MALDI-TOF MS	Vancomycin
6	De Frutos, M., et al., 2018 [39]	46-year-old male	Right otalgia with suppuration	Right ear	Pure culture	Oral and topical ciprofloxacin
7	De Frutos, M., et al., 2018 [39]	2-year-old, male	Left otitis media	Left ear	Otic smear, culture	Amoxicillin
8	De Frutos, M., et al., 2018 [39]	28-year-old, female (21 weeks pregnant)	Episodes of otitis	Ear	Culture	Topical beclomethasone and clioquinol
9	De Frutos, M., et al., 2018 [39]	9-year-old, female	Suppurative otitis media with left ear pain	Left ear	Smear and Culture	Amoxicillin-clavulanic acid and topical ciprofloxacin
10	De Frutos, M., et al., 2018 [39]	13-month-old, male	Erythema in the left retroauricular area and detachment of the auricular pavilion	Ear exudate	Culture	Meropenem and oral amoxicillin-clavulanic acid
11	Halle, T.R., N.W. Todd, and J. Fainberg, 2017 [18]	10-month-old female	Recurrent acute otitis media	Right ear	Culture	Ciprodex-otic drops, bilateral cochlear implantation
12	Bîrluţiu, V et al., 2017 [12]	75-years-old, male	Spastic paraplegia, and confusion, altered general condition	Blood culture	API® Coryne and culture	Fosfomycin
13	Gaona, C.E. and J.S. Castañón, 2017 [25]	6 year old, female	Otitis externa	Ear exudate	Gram stain, media growth, API Coryne, Vitek MS system	Ciprofloxacin ear drops
14	Gaona, C.E. and J.S. Castañón, 2017 [25]	53-year old, male	Right otorrhea with purulent discharge	Right ear	Gram stain, media growth, API Coryne, Vitek MS system	Tobramycin ear drops
15	Johnson, A.K. and B. Isaacson., 2016 [17]	4-year-old, male	Progressive right post-auricular erythema, otalgia, fever, and vomiting	Purulent middle ear effusion	Culture	Intravenous antibiotics
16	MA, S.C. 2014 [41]	14-month-old female	Fever, retroauricular edema with detachment of the left pinna.	Otic exudate	Culture	Cefotaxime and Amoxicillin-clavulanic acid
17	Ježek, P., et al., 2011 [29]	4 year old, male	Otitis media and spontaneous tympanic membrane perforation	Left ear	Rapid CB Plus kit, MALDI MS, culture, biochemicals	Beta-Lactam
18	Jeziorski, E., et al.2009 [21]	3 years and 3 months old, female	Acute perforated otitis media complicated by mastoiditis	Spontaneous otorrhoea	Smear and culture	Cefotaxime, vancomycin, amoxicillin–clavulanic acid
19	Poulter, M.D. and C.J. Hinnebusch, 2005 [35]	23-year-old, male	Right tympanic membrane retracted with a middle ear effusion	Middle ear effusion	Smear, culture, API Coryne system, and biochemicals	Augmentin
20	C. Loiez et al., 2002 [34]	10-year-old, male	Acute lymphoblastic leukemia B	Blood culture and ear swab	API Coryne system, culture, CAMP test	Oral amoxicillin
21	Dana, A., R. Fader, and D. Sterken, 2001 [2]	5-year-old, female	Bilateral ear pain	Right and left middle	Gram Stain, API Coryne, culture	Intravenous cefotaxime
22	Reynolds, S.J., M. Behr, and J. McDonald, 2001 [3]	3-year-old, female	Pain and swelling behind her right ear	Ear	PCR, culture, CAMP test, High-performance liquid chromatography analysis	Intravenous cefuroxime and cloxacillin
23	Fernandez Perez, A., et al. 1996 [13]	7-year-old, male	Upper right neck pain radiating to the mastoid region	Cervical abscess	API Coryne and culture	NS
24	Renaud, F.N., et al. 1996 [1]	6-month-old, female	Bilateral maxillolabiopalatine cleft with mucopurulent discharge	Ear	API Coryne, culture and biochemicals	Amoxicillin-clavulanic acid

**Table 2:** Shows the summary of retrospective studies of patients with *T. otitidis* infection

No.	Author and year	Age	Sample size	No. of *Turicella otitidis* cases or percentage	Condition	Source of isolation	Identification		
1	Gavrilovici, C., et al., 2022 [9]	2 months-7 years	147	*n* = 1	Acute otitis media	Pus	MICROSCAN panels (PC42, HIND) and MALDI-TOF MS	NA	
2	Mendez-Legaza, J.M., et al.,2021 [23]	0–14 years and 2–81 years	1089 samples	*n* = 22	ear exudate	External auditory canal	Culture, API Coryne, MALDI-TOF MS		
3	Chen, T.Y., et al. 2021 [20]	NS	107 culturable isolates	*n* = 10	Otitis media	Middle ear sample	NS		
4	Álvarez, A.S. and M.G. Coca, 2021 [32]	NS	273 ear exudates	*n* = 18	Acute otitis media	Middle ear fluid samples	ANC card of the Vitek2® system		
5	Barron, C.L., et al., 2020 [19]	9 months- 19 years	38 effusion	*n* = 1	Chronic middle ear effusions	Middle ear fluid	Culture, Immunoblot (DNABII proteins)		
6	Ari O., et al., 2019 [15]	NS	25 children	6%	Otitis media with effusion	Ear	16 S rRNA metagenomics		
7	Man, W.H., et al., 2019 [28]	> 5 years	94 children	*n* = 5	Acute otitis media	Tympanostomy tube otorrhea	16 S ribosomal RNA-based sequencing		
8	Kolbe, A.R., et al., 2019 [10]	3-176 months	50 children	*n* = 26	Chronic otitis media	Middle ear fluid	DNA sequencing		
9	Kalcioglu, et al., 2018 [24]	NS	102 samples	*n* = 5/26 (cholesteatomy), *n* = 4/18 (typanosclerotic plaque	Chronic otitis media	Middle ear	PCR, 16 ribosomal RNA		
10	Sillanpää, S., et al., 2017 [42]	5–42 months	79	*n* = 5	Acute otitis media	Middle ear fluid samples	Nested-PCR amplification of the 16 S rRNA gene (V4 region), mass sequencing		
11	Vila, P.M., et al., 2017 [22]	2–6 years	54	*n* = 4	Otitis media (cochlear implant)	Middle ear	Culture		
12	Krueger, A., et al., 2017 [27]	0–24 months (n = 25), > 24 month (n = 30)	55	7.84%	Middle ear effusion	Middle ear	Western blot and DNA and molecular analysis		
13	Quesnel, S., et al., 2010 [8]	3 months-15 years	188	*n* = 4	Acute mastoiditis	Drainage of retro auricular abscess, paracentesis	Culture		
14	Jeziorski, E., et al.2009 [21]	NS	12	*n* = 12	Acute otitis media, acute pyelonephritis, seromucous otitis, acute myeloid leukemia, acute lymphoid leukemia	Middle ear	Stain, culture, DNAse, CAMP test		
15	Gomez-Garces, J.L., et al., 2004 [43]	6 months-7 years	153 ear exudates (112 patients)	*n* = 7	Acute exudative otitis media or exacerbation of chronic otitis media	Middle ear	API Coryne, API Zym, API 50 CH, morphological and phenotypic tests		
16	Holzmann, D., et al., 2002 [30]	NS	60 children	*n* = 14	Exudative otitis media	Middle ear effusion	Culture, biochemical and chemotaxonomic data		
17	Funke, G., et al., 1994 [39]	1–5 years	NS	NS	Otitis media	Middle ear fluid	Biochemical, API Coryne, and 16 S rRNA sequencing		

*NS* Not specified, *PCR* Polymerase chain reaction

We herein provide a summary of the genome and cellular fatty acid analysis of *T. otitidis* draft strain (TD1) isolated from a central line catheter tip culture in a patient with a history of bowel obstruction and ATCC 51,513 strain from the ear [35, 44]. Comparative studies were conducted to investigate the changes in the genome and metabolic pathways of both strains. Upon comparing the identity of TD1 to ATCC 51,523 (with a similarity of 98.75%), Greninger et al. performed genome analysis which showed that TD1 has a size of 2,150,112 bp with an *N*_50_ of 24,176 bp and a GC content of 71.2%. On the other hand, ATCC 51,513 has a size of 2,077,086 bp with an average G + C content of 71.35%. Direct mapping of TD1 reads to ATCC 51,513 revealed 20,176 variants between the two strains. Furthermore, the TD1 strain contains 85.4 kb unique sequences when compared to ATCC51513. Notably, this includes the locus of 50 kb that contains over 60 hypothetical protein-coding sequences. In TD1 strain, various genetic variants in the genome exist, which encompass exons coding for an arylsulfatase, a cadmium-cobalt antiporter, a phosphate/phosphonate transporter operon, and a unique ATP-binding cassette (ABC) transporter. These findings imply a potential role in the survival and adaptation of *T. otitidis* to diverse niches. Also, TD1 strain has antibiotic resistance genes, including a cfrA 50 S methyltransferase that shares 99% amino acid similarity with *T. otitidis* ATCC 51,513, as well as two major facilitator superfamily–type drug-resistance transporters that exhibit 99% amino acid similarity to *T. otitidis* ATCC 51,513 and 57–59% amino acid similarity to *Corynebacterium* species [35, 44].

## Discussion

**Fig. 2:**
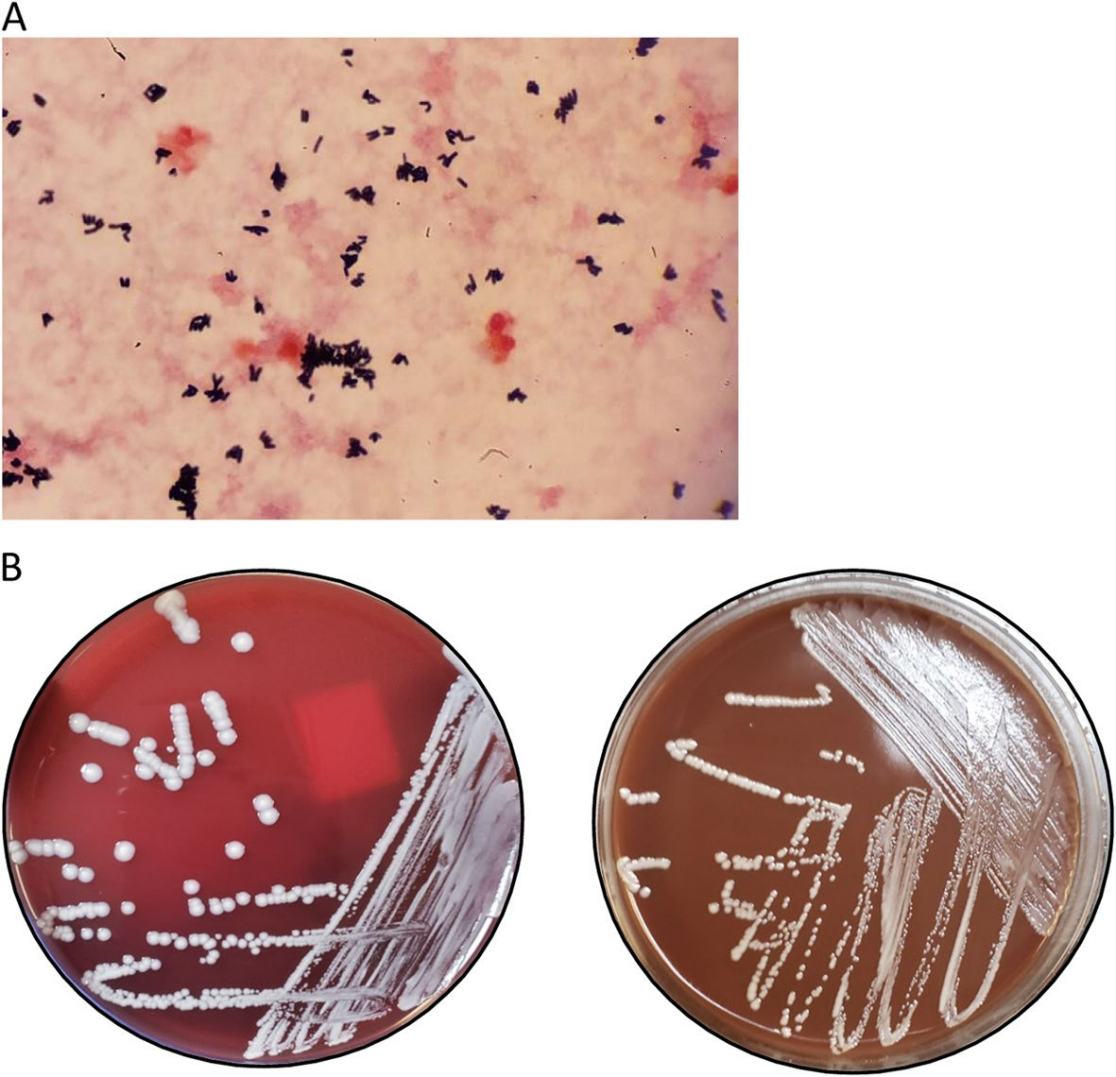
**A** shows pleomorphic Gram-positive rods, a characteristic morphology of *Corynebacterium,* “Chinese letters”. **B** shows the growth of non-pigmented white colonies of *T. otitidis* on blood and chocolate agar inoculated with a positive blood culture incubated for 24 hours at 35 ^O^C

*Turicella otitidis* is a constituent of the normal ear microbiota [40] and has been linked to both acute and chronic otitis media [30]. *T. otitidis* is a non-branching, long, irregular, Gram-positive bacillus that forms non-hemolytic, creamy/whitish colonies after 24 h of incubation (Fig. 2A and B). The organism is negative for catalase, alkaline phosphatase, and nitrate reduction. However, it is positive for the CAMP test, leucine arylamidase, and pyrazinamidase. Additionally, it is non-motile and urease negative. The ultimate differentiation of *Corynebacterium* species is based on conventional diagnostics [1] together with MALDI-TOF MS. Recently, the identification of *T. otitidis* from extra-otic sources suggests the adaptability of the microorganism and its potential to cause significant extra-otic infections in susceptible individuals. Thus necessitating the appropriate speciation of *Corynebacterium* when recovered from sterile sites to ensure that patients receive optimal and timely treatment [41].

Human immune system is a multi-faceted confluence of intricate cellular and biochemical responses. Microorganisms react to the dynamic immune milieu by disrupting host defense mechanisms and adapting to the environment to thrive and propagate. To evade eradication by the host immune response and subsist in the new environment, bacteria employ various strategies such as genome alteration and changes to their metabolic profile [45]. In this study, we summarized genome and cellular fatty acid analysis of *T. otitidis* draft strain (TD1) isolated from a central line catheter tip and ATCC 51,513 strain isolated from the ear.

Unlike ATC5153, TD1 strain possesses genes for various transporters such as arylsulfatase. Arylsulfatases are enzymes that break down aryl sulfonate ester bonds to release sulfonate, a source of sulfur synthesis required in the biosynthesis of cysteine and methionine. Arylsulfatases play a role in redox reactions and are linked to bacterial pathogenesis [46]. TD1 strain also contains a cadmium-cobalt antiporter. This anti-porter helps to regulate bio-metal levels, which are important for metabolic processes and are inserted into specific metalloproteins during biosynthesis. Microorganisms adapt to environmental pressure by importing essential metals while preventing excessive accumulation and intoxication through transporters and anti-transporters [43], suggesting a similar role of these transporters in *T. otitidis*. In addition, TD1 possesses phosphate/phosphonate transporter operon and a unique ABC transporter. Phosphate is a crucial nutrient found as phosphorus in nature. Bacteria have diverse transport systems, including a high-affinity phosphate-specific unique ABC transporter, to ensure phosphorus availability. They also have a system for alternative phosphorus sources [47–49]. However, the role of these transporters in *T. otitidis* pathogenesis is not fully understood.

Microorganism’s distinctive metabolic characteristics are linked to its natural habitat. Like *T. otitidis*, which encodes for a four-step histidine utilization pathway that facilitates the conversion of L-histidine to L-glutamate [50]. The low concentration of histidine in the middle ear serves as a limiting factor for pathogenic bacteria that cause otitis media. The metabolism of glutamate plays a crucial role in providing resistance to bacterial stress responses. Both TD1 and ATC5153 possess the gene responsible for converting histidine to glutamate, enabling pathogenic *T. otitidis* to overcome the limiting factor as well as a stress response. This supports the colonization of *Turicella otitidis* in the middle ear, leading to otitis media, whereas other bacteria are unable to colonize and cause infection [44].

*Turicella otitidis* harbors genes for selenocysteine synthesis, which is incorporated into selenoproteins, in addition presence of nucleophilic amino acids and modulation of redox potential, suggests that *T. otitidis* is highly adaptable to environmental conditions, which enables it to cause extra-otic infections [41, 51]. Moreover, *T. otitidis* is capable of catabolizing taurine, a sulfur-containing β-amino acid that plays significant roles in antioxidative and anti-inflammatory reactions and promotes the immune defense against microbial infections by enhancing the metabolism and functions of immune cells such as monocytes and macrophages [51, 52]. *T. otitidis* lacks essential genes, namely *mabA, inhA, kasA*, and *hadB* (Fatty Acid Synthesis (FAS-II) pathway), which are involved in the biosynthesis of fatty and mycolic acid [52]. In addition, the fatty acid profile shows that *T. otitidis* possesses unsaturated menaquinones (MK-10 and MK-11) as opposed to dihydrogenated menaquinones (MK-8(H2) and MK-9(H2)). Menaquinone (Vitamin K2) is an essential, electron carrier intricately involved in anaerobic redox reactions leading to ATP generation. Furthermore, *T. otitidis* lacks mycolic acid, and this absence plays a role in preventing cell wall permeability, thereby imparting resistance to antibiotics and phagocytosis, unlike other forms of Corynebacterium species [37]. As a result of niche-specific changes in the genome and metabolism of a bacterium, the mechanism of adaptability and pathogenicity is suggested to be linked to the immune status of the host. Several retrospective studies have demonstrated that *Turicella otitidis* is a prevalent cause of ear infections in the pediatric population [22, 53].

Antibiotics function by utilizing the virulence factors and anatomical features of a pathogen to disable its reproductive ability (bacteriostatic) or eradicate it (bactericidal). The resistance of *Turicella otitidis* to macrolides and lincosamides can be attributed to the presence of 23 S rRNA mutations [54]. The genome sequence of *Turicella otitidis* both strains, TD1 and ATCC 5153 possess a cfrA 50 S methyltransferase and two major facilitator superfamily-type drug resistance transporters. Given that *T. otitidis* is regarded as an emerging pathogen, it is of utmost importance to closely monitor the potential development of resistance in the future. Recent research has demonstrated that *Turicella otitidis*, in conjunction with other microorganisms, demonstrates resistance to high concentrations of ototopical antibiotics, including ciprofloxacin [5]. However, there is currently insufficient data available on *Turicella otitidis* resistance to the minimum inhibitory concentration (MIC) breakpoint, except in the presence of clindamycin and macrolides [55]. Although studies on otitis media have demonstrated the recurrence of infection when *T. otitidis* is not treated with appropriate antibiotics, there have been no reported occurrences of bacteremia or other invasive infections by *Turicella otitidis*. The organism has been reported to exhibit high susceptibility to beta-lactam antibiotics such as penicillin, cephalosporins, and carbapenems, as well as to chloramphenicol, linezolid, vancomycin, and teicoplanin. In all summarized cases, administration of broad-spectrum antibiotics showed successful recovery of patients infected with *Turicella otitidis*. However, the virulence factors of *Turicella otitidis* and its potential to cause extra-otic infections remain to be fully understood. One limitation of our study is the sparse number of cases available on *Turicella otitidis*. Though the genomic and metabolic analysis from two accessible studies has been summarized here, additional information is required to make conclusions about the observed variability in the genome and metabolic profile. Moreover, it is necessary to ascertain whether this information can be extrapolated to *Turicella otitidis* isolates from otitis media and extra otic infections in patients with or without underlying conditions, furthermore, the role of the host immune response in the adaptability and pathogenicity of the organism remains to be characterized in detail.

## Summary

*Turicella otitidis* is a coryneform bacteria commonly associated with both acute and chronic otitis media. However, recent reports of extra-otic infections caused by *Turicella otitidis* and the emergence of antimicrobial resistance are concerning as the organism can cause infection in vulnerable populations. Despite this, our current understanding of the pathogenicity of *Turicella otitidis* remains limited. This study summarizes the metabolic and genomic characteristics of two isolates of *T. otitidis* strains from two different sources, suggesting that the bacterium is capable of shaping its environment and undergoes alterations in metabolic pathways, gene transcription, and proteome composition. These findings offer valuable insights into the metabolic pathophysiology of the bacterium, enabling it to adapt and survive in diverse niches.

### Supplementary Information

**Additional file 1: Electronic Supplementary file S1.** Literature search summary (1994-2023).

## Data Availability

All data generated or analyzed during this study are included in this published article.
