# Supplementary material for: Metabolism and gene sequence variation in Turicella otitidis implies its adaptability and pathogenicity in extra-otic infection: a systematic review
Source: BMC Infect Dis. 2023 Oct 27;23:735. doi: 10.1186/s12879-023-08721-y (PMC10612267; doi:10.1186/s12879-023-08721-y)
Supplement: Supplementary file 1 — Additional file 1: Electronic Supplementary file S1. Literature search summary (1994-2023). [file 12879_2023_8721_MOESM1_ESM.docx]

# Electronic Supplementary file, S1: Literature search summary (1994-2023)

Databases (PubMed, Cochrane, EMBASE, CINAHL Plus with Full Text CINAHL, Science Direct, and Google Scholar) were searched from 1994-2023.

| Name of the databases | Search terms and results |
| --- | --- |
| PubMed (n=87) | #1“Turicella otitdis”, OR [MeSH] 42  #2T. otitidis OR [MeSH] 26  #3 Turicella otitdis metabolism OR [MeSH] 3  #4Turicella otitdis metabolism OR [MeSH] 5  #5Turicella otitidis genome sequence OR [MeSH] 5  #6T. otitidis genome sequences OR [MeSH] 5  #7Turicella otitidis metabolism and genome sequence OR [MeSH] 1 |
| Cochrane (n=0) | #1 ("Turicella otitidis"):ab (Word variations have been searched) in Cochrane Reviews, Cochrane Protocols, Trials, Clinical Answers, Editorials, Special Collections 0  #2 Turicella otitidis 0  #3 T. otitidis 0  #4 Turicella otitidis metabolism 0  #5 Turicella otitidis genome sequence 0  #6 T. otitidis metabolism 0  #7 T.otitidis genome sequence 0 |
| EMBASE (n=52) | #1'turicella otitidis'/exp OR 'turicella otitidis' 48  #2 turicella AND otitidis AND metabolism [3](https://www.embase.com/)  #3 turicella AND genome AND sequences [0](https://www.embase.com/)   - #4 turicella AND otitidis AND metabolism AND genome AND sequence [1](https://www.embase.com/) |
| CINAHL Plus (n=30) | Search modes - Boolean/Phrase Interface - EBSCOhost Research Databases  S1 Turicella otitids 6  S2T. otitidis 3  S3 Turicella otitidis genome sequences 0  S3 Turicella otitidis metabolism 6  S4 T. otitids metabolism 4  S5 T. otitidis genome sequences 4  S6 Turicella otitidis genome sequences 1  S7 Turicella otitidis metabolism and genome sequence 6 |
| Science Direct (n=289) | Term(s): "Turicella otitidis" OR "T. otitidis" 96  Turicella otitidis metabolism" 21  Turicella otitidis genome sequence" 22  T. otitidis metabolism" 69  T. otitidis genome sequence" 71  Turicella otitidis metabolism and genome sequence 10 |
| Google Scholar (n=4351) | Turicella otitidis" \|"T. otitidis" 508  Turicella otitidis metabolism 197  T. otitidis metabolism 1300  Turicella otitidis genome sequences 346  T otitidis genome sequences 1830  "Turicella otitidis metabolism genome sequences" 170 |
